# Normal thromboelastography with a markedly prolonged activated partial thromboplastin time in severe prekallikrein deficiency: Implications for perioperative hemostatic assessment

**DOI:** 10.1111/trf.70327

**Published:** 2026-07-15

**Authors:** Jeremy W. Jacobs, Anton Matafonov, Patricia G. Baxter, Qiufang Cheng, Garrett S. Booth, Deva Sharma, Benjamin F. Tillman, David Gailani

**Affiliations:** ^1^ Department of Pathology, Microbiology, and Immunology Vanderbilt University Medical Center Nashville Tennessee USA; ^2^ Esoteric Coagulation Laboratory Vanderbilt Medical Laboratories Nashville Tennessee USA; ^3^ Division of Hematology/Oncology, Department of Medicine Vanderbilt University Medical Center Nashville Tennessee USA

**Keywords:** activated partial thromboplastin time, coagulopathy, contact activation, prekallikrein deficiency, preoperative evaluation, TEG 6s, thromboelastography

## Abstract

**Background:**

Prekallikrein (PK) deficiency is a rare contact factor deficiency that can markedly prolong the activated partial thromboplastin time (aPTT) without a bleeding phenotype. Because viscoelastic hemostatic assays also rely on contact activation, their behavior in severe PK deficiency is relevant to perioperative evaluation of isolated aPTT prolongation. We describe thromboelastography (TEG) 6s findings in a patient with severe PK deficiency.

**Study Design and Methods:**

A 59‐year‐old woman without a bleeding history was evaluated for an isolated prolonged aPTT identified during preoperative testing. Studies included prothrombin time (PT), thrombin time, an anti‐factor Xa assay, aPTT mixing studies including with PK‐deficient plasma, and factor VIII, IX, XI, and XII activities. PK antigen was assessed by western immunoblotting. Whole blood viscoelastic testing was performed on a TEG 6s analyzer.

**Results:**

The patient had a markedly prolonged aPTT (87.4 s, reference range [RR]: 24.5–34.0 s) with normal PT, thrombin time, and a negative anti‐factor Xa assay. The aPTT corrected on mixing with normal pooled plasma but not with PK‐deficient plasma. Factor VIII, IX, XI, and XII activities were normal or modestly elevated. Western immunoblotting showed absent PK antigen, establishing cross‐reactive material‐negative PK deficiency. Despite the prolonged aPTT, TEG 6s parameters were normal, including the citrated kaolin R time (4.6 min, RR: 4.6–9.1 min). The patient underwent surgery without hemostatic complications or transfusion.

**Discussion:**

Severe PK deficiency may markedly prolong the plasma‐based aPTT without parallel abnormalities on TEG 6s, highlighting assay‐specific differences in sensitivity to PK deficiency.

**Abbreviations**
aPTT: activated partial thromboplastin time
CFF: citrated functional fibrinogen
CK: citrated kaolin
CRM: cross‐reactive material
CRT: citrated Rapid TEG
ELISA: enzyme‐linked immunosorbent assay
FIX: factor IX
FXI: factor XI
FXII: factor XII
FVIII: factor VIII
HK: high‐molecular‐weight kininogen
HRP: horseradish peroxidase
LY30: lysis at 30 minutes
MA: maximum amplitude
PT: prothrombin time
ROTEM: rotational thromboelastometry
RR: reference range
TEG: thromboelastography
VHA: viscoelastic hemostatic assay

## 1 INTRODUCTION

Contact activation is initiated when blood plasma encounters negatively charged surfaces. The plasma contact system is comprised of the protease precursors factor XII (FXII), prekallikrein (PK), and factor XI (FXI), and the cofactor high‐molecular‐weight kininogen (HK).^1^ The proteins assemble and become activated on the charged surface, generating proteolytic activity that promotes blood coagulation.^2^, ^3^ On the surface, FXII initially undergoes a conformational change leading to autocatalytic conversion to the active protease FXIIa (Figure 1, reaction 1). PK and FXI circulate in plasma predominantly as complexes with the cofactor HK. PK is converted to kallikrein by FXIIa, and kallikrein in turn activates additional FXII, amplifying the contact process (Figure 1, reactions 2 and 3).^2^, ^3^, ^4^ FXIIa also converts FXI to FXIa, which promotes plasma coagulation by activating factor IX (Figure 1, reaction 4). During contact activation, HK facilitates PK and FXI binding to surfaces, enhancing their activation by FXIIa.^5^, ^6^

**FIGURE 1:**
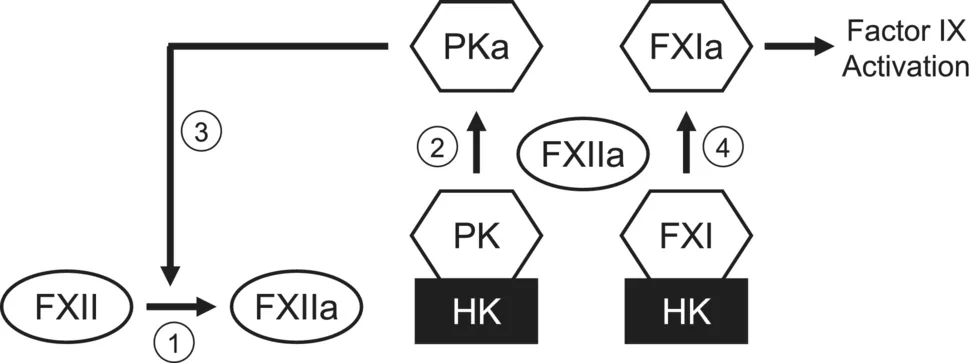
Contact activation. Shown is a schematic diagram of plasma reactions occurring on a negatively charged surface during contact activation. The process is initiated by autocatalytic conversion of factor XII (FXII) to the active protease FXIIa (reaction 1). FXIIa then converts prekallikrein (PK) to kallikrein (PKa) in a reaction enhanced by the cofactor high‐molecular‐weight kininogen (HK) (reaction 2). PKa amplifies contact activation by converting additional FXII to FXIIa (reaction 3). FXIIa also converts FXI, a homolog of PK, to the protease FXIa (reaction 4), which promotes clot formation by activating factor IX in the presence of calcium ions.

Contact activation drives fibrin clot formation in the activated partial thromboplastin time (aPTT) assay used in clinical practice, and a deficiency of any one contact factor significantly prolongs the aPTT. Yet, despite the important roles for FXII, PK, and HK in this process, individuals lacking one of these proteins do not experience abnormal bleeding. Of the contact factors, only FXI deficiency is associated with abnormal hemostasis. This is because hemostasis in vivo is initiated primarily by the factor VIIa/tissue factor complex and not by contact activation.^7^ Accordingly, FXII, PK and HK deficiencies form a group of conditions that produce striking abnormalities in the aPTT without corresponding hemorrhagic manifestations. Identification of these conditions is important to distinguish them from deficiencies of factor VIII (FVIII), factor IX (FIX), and FXI, which are associated with bleeding.

PK deficiency results from pathogenic mutations in, or deletions of, the *KLKB1* gene and is inherited in an autosomal recessive manner.^8^ Severe PK deficiency encompasses both cross‐reactive material‐positive (CRM+) and cross‐reactive material‐negative (CRM−) forms.^8^, ^9^, ^10^ In CRM+ deficiency, dysfunctional PK protein is immunologically detectable in the plasma, whereas CRM− deficiency is characterized by an absence or reduction in protein level (Figure 2A). Because functional assays do not distinguish severe dysfunction from protein absence, definitive classification of the PK‐deficiency phenotype requires assessment of PK antigen by western immunoblotting or enzyme‐linked immunosorbent assay (ELISA).

**FIGURE 2:**
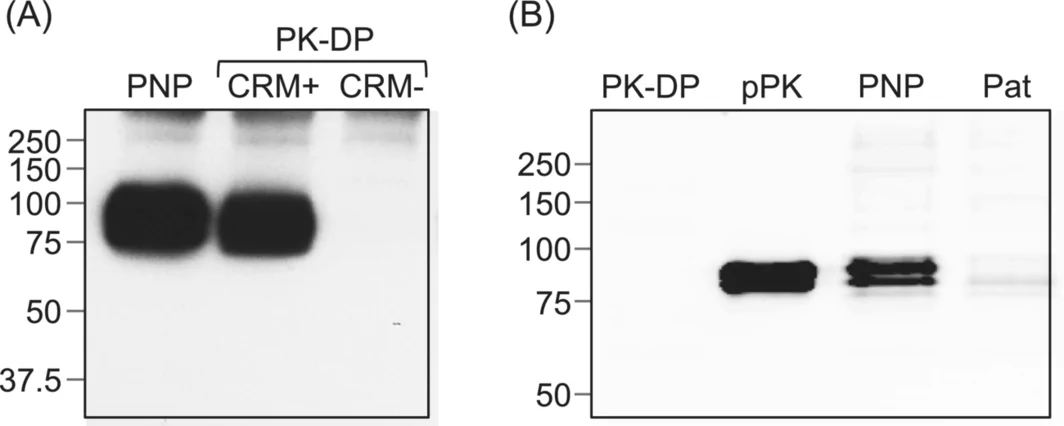
Western blots of plasma. (A) Western immunoblot of pooled normal plasma (PNP), plasma from an individual with cross‐react material‐positive prekallikrein (PK)‐deficiency (PK‐DP CRM+), and plasma from an individual with cross‐react material‐negative PK‐deficiency (PK‐DP CRM−). (B) Western immunoblot of CRM− PK‐deficient plasma (PK‐DP), purified human plasma PK (pPK), PNP, and patient plasma (Pat). For the blots in panels (A) and (B), PK was detected with an horseradish peroxidase (HRP)‐conjugated polyclonal IgG to human PK and chemiluminescence. In both panels, positions of molecular mass markers in kiloDaltons are shown on the left.

Viscoelastic hemostatic assays (VHAs), including thromboelastography (TEG) and rotational thromboelastometry (ROTEM), provide assessment of initiation of clot formation, clot strength, and clot degradation in whole blood.^11^ Similar to the aPTT assay, VHAs often induce contact activation to initiate coagulation. In patients with unexplained isolated aPTT prolongations, particularly in perioperative settings where rapid clarification of bleeding risk is often needed, understanding the behavior of VHA platforms in contact factor deficiencies has practical relevance. Here, we describe a patient with CRM− PK deficiency in whom results of TEG 6s testing were normal despite a markedly prolonged aPTT and discuss the mechanistic and clinical implications of this dissociation.

## 2 MATERIALS AND METHODS

### 2.1 Coagulation assays

Platelet‐poor plasma was prepared from normal donor and patient blood collected into 1/10th volumes of 3.2% sodium citrate. Plasma aPTTs were measured on a STA‐R MAX analyzer (Diagnostica Stago, Parsippany, NJ) using STA‐PTT Automate 5 reagent per the manufacturer's instructions. The prothrombin time (PT), thrombin time, fibrinogen level (Clauss method), an assay for factor Xa inhibitors, and individual coagulation factor activities (for factors VIII, IX, XI, and XII) were determined on the same platform. PK deficiency was confirmed by performing an aPTT on a 1:1 mix of patient plasma and PK‐deficient plasma (George King Bio‐Medical, Overland Park, KS, USA). Failure of the aPTT to correct into the normal range indicates the patient's plasma has a low level of PK activity.

### 2.2 TEG 6s analysis

Whole blood collected into a 1/10th volume of 3.2% sodium citrate was analyzed on a TEG 6s analyzer (Haemonetics, Boston, MA) using the Global Hemostasis with Lysis cartridge per the manufacturer's instructions. The citrated kaolin (CK) channel containing a negatively charged surface (kaolin) induces clotting through contact activation in a whole blood matrix. The citrated rapid TEG (CRT) channel uses both kaolin and recombinant tissue factor to accelerate clot initiation. The citrated functional fibrinogen (CFF) channel uses tissue factor in the presence of the platelet blocker abciximab to estimate the contribution of fibrinogen to clot strength. Parameters recorded included R time (time to initial clot formation with a 2 mm amplitude), maximum amplitude (MA, in millimeters), and percent lysis at 30 min (LY30). Institutional reference ranges were used for interpretation.

### 2.3 Western immunoblotting

Citrated plasma from two individuals with severe PK deficiency was purchased from George King Bio‐Medical, Inc. (Overland Park, Kansas, USA). One microliter of pooled normal plasma, the PK‐deficient control plasmas, patient plasma, and a 2 μg sample of pure PK (Enzyme Research Lab., South Bend, IN, USA) were each diluted with 9 μL of phosphate‐buffered saline, and non‐reducing SDS‐sample buffer was added. Plasmas were size‐fractionated on 10% polyacrylamide‐SDS gels and transferred to nitrocellulose membranes. Western blots were developed with horseradish peroxidase (HRP)‐conjugated sheep anti‐human prekallikrein IgG (Affinity Biologicals, Ancaster, Ontario, Canda) and chemiluminescence.

## 3 CASE REPORT

A 59‐year‐old female with no prior history of bleeding was scheduled to undergo abdominal surgery for evaluation of a pancreatic mass. She was not taking anticoagulant or antiplatelet medications. Routine preoperative laboratory testing revealed a prolonged aPTT (87.4 s, reference range [RR]: 24.5–34.0 s), a normal PT (13.2 s, RR: 12.4–15.0 s), and a normal platelet count. The results were confirmed with a second blood sample. The thrombin time was normal and the anti‐factor Xa assay was negative, excluding heparin, direct thrombin inhibitors, and direct factor Xa inhibitors as causes of the prolonged aPTT. The fibrinogen was moderately elevated at 582 mg/dL (RR: 188–450 mg/dL). A 1:1 mixing study of patient plasma with pooled normal plasma produced immediate and complete correction of the aPTT that persisted through 2 h of incubation (0‐h: 26.7 s; 1‐h: 27.7 s; 2‐h: 28.8 s), indicating a factor deficiency and arguing against a circulating inhibitor. Activity levels of factors VIII (198%), IX (148%), XI (174%), and XII (151%) were all at the upper end of their respective normal ranges or modestly elevated. The patient's prolonged aPTT failed to correct on mixing with PK‐deficient plasma, indicating the patient's plasma lacked PK.

Western immunoblotting for PK was performed on the patient's plasma and reference plasmas. The two immunoreactive bands representing normal PK migrate at ~85–88 kDa. The blot in Figure 2A demonstrates that a polyclonal anti‐PK IgG recognizes PK in pooled normal plasma (PNP) and in plasma from an individual with CRM+ PK deficiency, but not in plasma from an individual with CRM− PK‐deficiency. Figure 2B demonstrates that the patient's plasma (Pat) contains, at most, a trace of PK relative to the normal control (PNP) and a sample of pure PK (pPK), indicating the patient has a CRM− PK deficiency.

Viscoelastic testing using the TEG 6s analyzer demonstrated clot formation kinetics that were within institutional reference ranges across all channels (Table 1). Notably, the CK channel R time, the parameter most analogous to the aPTT, was normal (4.6 min, RR: 4.6–9.1 min).

**TABLE 1:** Coagulation parameters in the index patient.

Test	Patient value	Reference range
Plasma coagulation screening (Encounter 1)		
PT (s)	13.2	12.4–15.0
aPTT (s)	87.4	24.5–34.0
Plasma coagulation screening (Encounter 2)		
aPTT (s)	76.8	24.5–34.0
Comprehensive coagulation evaluation (Encounter 3)		
aPTT (s)	74.1	24.5–34.0
Thrombin time (s)	18	15–25
Anti‐factor Xa assay (IU/mL)	<0.10	<0.10
Fibrinogen (mg/dL)	582	188–450
aPTT mixing study (1:1 patient plasma + pooled normal plasma)		
0 h (immediate) (s)	26.7	24.5–34.0
1 h incubation at 37°C (s)	27.7	24.5–34.0
2 h incubation at 37°C (s)	28.8	24.5–34.0
Factor activity assays		
Factor VIII (%)	198	50–150
Factor IX (%)	148	50–150
Factor XI (%)	174	50–150
Factor XII (%)	151	50–150
Prekallikrein cross‐correction study (1:1 patient plasma + PK‐deficient plasma)		
Patient plasma alone (s)	43.9	—
PK‐deficient plasma alone (s)	50.8	—
1:1 mixture (s)	49.5	—
TEG 6s (whole blood viscoelastic testing)		
CK R time (min)	4.6	4.6–9.1
CK LY30 (%)	2.5	0–2.6
CRT MA (mm)	67.9	52–70
CFF functional fibrinogen MA (mm)	31.6	15–32

*Note*: Reference ranges for coagulation assays are institutional values (STA R MAX, Diagnostica Stago); TEG 6s reference ranges are institutional values (Haemonetics, Boston, MA).

Abbreviations: aPTT, activated partial thromboplastin time; CFF, citrated functional fibrinogen channel; CK, citrated kaolin channel; CRT, citrated rapid thromboelastography channel; LY30, lysis at 30 min; MA, maximum amplitude; PK, prekallikrein; PT, prothrombin time.

The patient and surgical team were counseled that PK deficiency is not associated with clinically significant bleeding and does not require perioperative replacement therapy. She subsequently underwent laparoscopic liver wedge biopsy, peritoneal washings, and right internal jugular Port‐A‐Cath placement without hemostatic complications (estimated blood loss 50 mL) and without special hemostatic precautions or transfusion support. Histopathologic assessment confirmed pancreatic adenocarcinoma.

## 4 DISCUSSION

We describe a 59‐year‐old woman with CRM‐negative PK deficiency whose TEG 6s analysis was normal despite a markedly prolonged aPTT. Although the TEG 6s CK channel and the aPTT rely on contact activation induced by a negatively charged substance to initiate clotting, the marked discordance between these assays indicates that they do not respond identically to severe PK deficiency. This distinction is clinically relevant because PK deficiency can produce striking abnormalities in plasma‐based clotting assays without a corresponding bleeding phenotype.

The aPTT is performed on citrated platelet‐poor plasma and is generally highly sensitive to defects in contact activation. In our clinical laboratory, the aPTT assay uses micronized silica (SiO_2_) to induce contact activation. Although FXII can undergo autoactivation on micronized silica (Figure 1, reaction 1) in the absence of PK, the assay is highly dependent on kallikrein activation of FXII (Figure 1, reaction 3) to generate sufficient FXIIa for adequate FXI activation. The absence of PK impairs this amplification loop and results in a substantial prolongation of the time to clot formation in this aPTT assay.^2^, ^4^ A characteristic feature of PK deficiency, which distinguishes it from deficiencies of FXII, FXI or HK, is the incubation effect, where prolonged incubation of PK‐deficient plasma with the silica‐based reagent before recalcification shortens or normalizes the aPTT.^12^ This occurs because the increased duration of incubation allows sufficient FXII autoactivation to compensate, at least partially, for the lack of PK. Thus, standard plasma‐based aPTT conditions accentuate the kinetic defect produced by PK deficiency.

In contrast to the aPTT, the CK channel of the TEG 6s uses whole blood and measures time to initial clot detection, defined by a 2 mm increase in amplitude. The CK channel uses kaolin, an aluminum silicate (Al_2_Si_2_O_5_ (OH)_4_), as the contact activator. Kaolin‐based systems are thought to be less sensitive to PK deficiency than systems using micronized silica‐based aPTT reagents.^13^ Furthermore, whole blood testing preserves cellular and platelet‐dependent surfaces that are absent from platelet‐poor plasma. These differences likely contributed to the normal CK R time observed in our patient. However, this finding should not be interpreted to mean that the TEG 6s CK channel is insensitive to contact pathway abnormalities in general. FXII is an absolute requirement for contact activation with any surface, and its absence will markedly prolong the CK channel R time just as it prolongs the aPTT. For example, Shrimpton et al. reported an R time of 38 min in the CK channel in blood lacking FXII.^14^ Similarly, HK not only supports PK activation but also FXI activation, and its absence causes a pronounced defect in contact activation regardless of the nature of the activating surface. Huang et al. reported prolonged R times on kaolin‐activated TEG tracings,^15^ and Jiang et al. described a markedly abnormal preoperative R time of 42.4 min due to HK deficiency.^16^ Therefore, the normal CK R time observed in this patient with PK deficiency should not be extrapolated to other contact factor deficiencies.

An important feature of this case is that the patient had pancreatic adenocarcinoma, a malignancy associated with a highly procoagulant phenotype, and tumor‐derived extracellular vesicles can promote FXII activation through surface‐exposed polyphosphate in a mouse model.^17^, ^18^, ^19^ We therefore cannot exclude the possibility that cancer‐associated contact pathway activation, elevated fibrinogen, platelet‐ or extracellular vesicle‐dependent surface assembly, or other whole blood procoagulant features contributed to the normal TEG 6s CK R time in this patient. However, severe PK deficiency would be expected to blunt kallikrein‐mediated amplification of FXII activation, and the patient's aPTT remained markedly prolonged despite the underlying malignancy. Accordingly, the patient's pancreatic cancer should be regarded as a potential confounder in interpreting the normal TEG 6s result, but it does not eliminate the observed assay discordance between the plasma‐based aPTT and whole blood TEG 6s testing. Testing additional whole blood samples from patients with severe PK deficiency, particularly individuals without active malignancy or other procoagulant conditions, would be needed to determine whether this finding is generalizable.

There are important practical implications from these findings. An unexplained isolated prolonged aPTT in the preoperative setting may prompt hematology consultation, delay procedures, and at times lead to unnecessary plasma transfusion.^20^ Deficiencies of FXII, PK, and HK are well‐recognized causes of isolated aPTT prolongation without a corresponding bleeding phenotype and do not require hemostatic treatment. In patients with PK deficiency, TEG 6s may remain normal despite a markedly prolonged aPTT, underscoring that these assays assess contact activation under different analytic conditions. A normal TEG 6s result should not be used to exclude PK deficiency or substitute for formal laboratory evaluation of an isolated aPTT prolongation. In a patient whose isolated prolongation is ultimately attributable to PK deficiency, however, a normal TEG 6s may offer supportive evidence of preserved hemostatic function while definitive laboratory classification is pursued.

## FUNDING INFORMATION

This work was supported by award HL140025 from the National Heart, Lung, and Blood Institute, and by the Ernest W. Goodpasture Chair in Experimental Pathology for Translational Research from Vanderbilt University.

## CONFLICT OF INTEREST STATEMENT

The authors have disclosed no conflicts of interest. Unrelated, DG is a consultant for several pharmaceutical companies with interests in developing drugs that target factor XI and prekallikrein. JWJ reports personal fees from Sanofi and Werfen, and research funding from Bayer and Diagnostica Stago.

## Data Availability

The data that support the findings of this study are available from the corresponding author upon reasonable request.
